# Seasonal risk of low pathogenic avian influenza virus introductions into free‐range layer farms in the Netherlands

**DOI:** 10.1111/tbed.13649

**Published:** 2020-06-07

**Authors:** Jose L. Gonzales, Sylvia Pritz‐Verschuren, Ruth Bouwstra, Jeanine Wiegel, Armin R. W. Elbers, Nancy Beerens

**Affiliations:** ^1^ Wageningen Bioveterinary Research (WBVR) Lelystad the Netherlands; ^2^ GD Animal Health Deventer the Netherlands

**Keywords:** Avian influenza, risk factors, seasonality, virus introduction

## Abstract

Poultry can become infected with avian influenza viruses (AIV) via (in) direct contact with infected wild birds. Free‐range chicken farms in the Netherlands were shown to have a higher risk for introduction of low pathogenic avian influenza (LPAI) virus than indoor chicken farms. Therefore, during outbreaks of highly pathogenic avian influenza (HPAI), free‐range layers are confined indoors as a risk mitigation measure. In this study, we characterized the seasonal patterns of AIV introductions into free‐range layer farms, to determine the high‐risk period. Data from the LPAI serological surveillance programme for the period 2013–2016 were used to first estimate the time of virus introduction into affected farms and then assess seasonal patterns in the risk of introduction. Time of introduction was estimated by fitting a mathematical model to seroprevalence data collected longitudinally from infected farms. For the period 2015–2016, longitudinal follow‐up included monthly collections of eggs for serological testing from a cohort of 261 farms. Information on the time of introduction was then used to estimate the monthly incidence and seasonality by fitting harmonic and Poisson regression models. A significant yearly seasonal risk of introduction that lasted around 4 months (November to February) was identified with the highest risk observed in January. The risk for introduction of LPAI viruses in this period was on average four times significantly higher than the period of low risk around the summer months. Although the data for HPAI infections were limited in the period 2014–2018, a similar risk period for introduction of HPAI viruses was observed. The results of this study can be used to optimize risk‐based surveillance and inform decisions on timing and duration of indoor confinement when HPAI viruses are known to circulate in the wild bird population.

## 1 INTRODUCTION

Avian influenza (AI) is a highly infectious viral disease that affects birds. AI viruses are classified into subtypes based on two surface glycoproteins, haemagglutinin (H) and neuraminidase (N). Sixteen H and nine N subtypes have been identified in birds, which can occur in different combinations. Low pathogenic AI (LPAI) viruses are naturally circulating in wild birds, and birds in the orders Anseriformes and Charadriiformes are considered the major virus reservoirs (Webster, Bean, Gorman, Chambers, & Kawaoka, 1992). Long‐distance migratory birds play a major role in the global spread of AI viruses (The Global Consortium for H5N8 & Related Influenza Viruses, 2016). Typically, LPAI infections in these birds are asymptomatic but can be associated with viral shedding in faeces. Domestic poultry can become infected with AI virus via the faecal–oral route, when poultry consume infectious faecal material from wild birds (von Waldburg‐Zeil, van Staaveren, & Harlander‐Matauschek, 2019). LPAI infections in poultry can cause mild clinical symptoms, leading to decreased egg production, decreased hatchability of eggs, misshapen eggs and mildly increased mortality (Gonzales & Elbers, 2018). LPAI viruses of the H5 subtype or H7 subtype can mutate to become highly pathogenic AI (HPAI) viruses in poultry, shortly after infection (Dietze et al., 2018) or after circulating undetected for longer times (Monne et al., 2014). However, since in 2005 the Asian HPAI H5N1 virus was detected in wild birds, HPAI H5 viruses were globally spread by wild bird migration (Nuñez & Ross, 2019). During the evolution of HPAI H5N1 viruses, reassortment events led to the generation of a range of novel subtypes. In the Netherlands, novel HPAI H5N8 reassortants caused outbreaks in poultry in 2014 and 2016 (Beerens et al., 2017; Bouwstra et al., 2015), and the novel HPAI H5N6 virus was introduced in 2017 (Beerens et al., 2018). HPAI viruses typically cause severe illness and high mortality in poultry. Every HPAI virus described has belonged to subtype H5 or subtype H7. Therefore, both HPAI and LPAI viruses of subtypes H5 and H7 are notifiable to the World Organisation for Animal Health.

In the Netherlands, outbreaks of H5 and H7 AI viruses are controlled by a ‘stamping out’ strategy, which involves quarantine and culling of all poultry on infected premises, tracing and surveillance of farms at risk and restriction of movement to reduce spread of the virus. Severe direct and indirect economical losses are associated with outbreaks of H5 and H7 viruses, as these affect international poultry trade (Longworth, Mourits, & Saatkamp, 2014). Measures taken during outbreaks may also include indoor confinement of free‐range poultry, to prevent contact between poultry and infected wild birds or a contaminated environment. When confinement lasts longer than the specified period of 16 weeks, the eggs cannot be commercialized as free range, leading to economic losses due to lower market price of non‐free‐range eggs.

The risk of outbreaks in commercial poultry has increased in recent years, due to circulation of HPAI H5 viruses in the wild bird population (European Food Safety Authority et al., 2019). Furthermore, the expansion of free‐range poultry farms, driven by consumers and animal welfare organizations, increases the opportunities for contact between poultry and wild birds (Bouwstra et al., 2017; Elbers & Gonzales, 2019). In the Netherlands, passive and active surveillance programmes are in place for the detection of AI viruses. In the active serologic surveillance programme, all poultry farms are tested at least once a year (30 random samples per flock; for laying hens at an age between 20 and approximately 84 weeks maximum; slaughter ducks: at the end of the production cycle, at an age of about 6–7 weeks; turkeys: at the end of the production cycle, at an age of about 16–20 weeks). Free‐range layer farms are sampled four times a year (30 random samples per flock; at an age between 20 and approximately 84 weeks maximum) based on their higher risk of AI introduction than the risk observed for farms that kept chickens indoors (Gonzales, Stegeman, Koch, de Wit, & Elbers, 2013). A previous analysis showed that the relative risk for introduction of LPAI on free‐range layer farms is 6.3‐fold significantly increased compared to farms with indoor housing (Bouwstra et al., 2017). Previous studies performed by Gonzales et al. (2013) and Bouwstra et al. (2017) did not find geographical clusters with high risk for introduction of LPAI in poultry within the Netherlands. It was shown, however, that farms located at short distances (<500 m) from water bodies or areas with high numbers of migratory wild birds had a higher risk of introduction than farms located at further distances (Bouwstra et al., 2017). Hence, the risks attributed to the poultry production system and geographical location have been quantified. However, the risk for introduction of AI in poultry farms may not be equal throughout the year due to factors such as the seasonal migration of wild birds (Verhagen et al., 2017) and possibly weather conditions. Information on the temporal risk of introduction will allow, together with the already identified risks, further optimization of risk‐based surveillance and control strategies.

The aim of this study was to characterize the seasonal patterns of AI virus introductions into free‐range layer farms using information obtained from the active serosurveillance programme for the years 2013 to 2016. Serological data were assessed retrospectively for the period 2013–2014. As for the period 2015 to 2016, in addition to the routine serological sampling, egg samples were collected and stored monthly and eggs collected from farms testing positive during routine serological surveillance were retrospectively analysed for antibodies against LPAI viruses. This enabled us to estimate the time of introduction of infection by fitting a mathematical model to the seroprevalence data of serum and eggs collected longitudinally from infected flocks, and these estimates were then used to assess the seasonal risk for introduction of infection by fitting harmonic and Poisson regression models. The implication of the findings of this study on the optimization of surveillance and the implementation of preventive measures are discussed.

## 2 METHODS

### 2.1 Study design and study population for the detection of LPAI virus introductions

The study population was all free‐range layer farms in the Netherlands during the period of 2013 (*n* = 453) to 2016 (*n* = 490). The study design was a combination of:

- A prospective study where a cohort of 261 farms, which voluntarily joined the study, were sampled monthly for the period between 2015 and 2016. Farms which were not part of the cohort were monitored, four times per year, as part of the surveillance programme (see Section 2.2).
- A retrospective study where the results of all surveillance tests made on all free‐range farms during the period 2013–2014 was retrieved for analysis.

Originally, only a prospective study was planned; however, at the end of the study the number of detected introductions was too low (see Section 3.1) to reliably assess seasonality. Therefore, it was decided to include the retrospective study.

### 2.2 Data sources used for the detection of LPAI virus introductions

We analysed all the serological data from the Dutch surveillance programme collected from free‐range layers during the study period 2013–2016. In this programme, all free‐range layer farms are sampled (30 random serum samples/farm) every 3 months and when a farm is confirmed seropositive, additional samples (serum and swabs) are taken to either confirm or exclude active infection (Bouwstra et al., 2017). Seropositive farms included in the analysis were only those considered as primary introductions. Farms likely infected by secondary farm‐to‐farm transmission, identified based on field and genetic observations (Bergervoet, Heutink, Bouwstra, Fouchier, & Beerens, 2019; Bouwstra et al., 2017), were excluded.

For the prospective study, 30 eggs per farm were collected monthly from April 2015 to November 2016 and stored for a period of maximum 6 months. If during routine surveillance a farm was detected seropositive, the stored eggs from the affected farm were tested retrospectively until a negative result was observed. All tests done using egg samples and serum samples (routine surveillance) allowed us to monitor changes in prevalence in time. It should be noted that this study started in April 2015 and finished in November 2016 because of the HPAI epidemics in 2014–2015 (Bouwstra et al., 2015) and 2016–2017 (Beerens et al., 2017). During these epidemic periods, there was a national obligation to keep chickens indoors. Hence, the egg sampling period includes those months when chickens were allowed outdoors.

For the retrospective study (2013–2014), only serological results were retrieved, which provided information on seroprevalence with 3‐month intervals. Exceptions were positive farms detected via the early warning programme (Bouwstra et al., 2017). When LPAI was detected via this programme, data on serological tests performed at detection and previous surveillance results were retrieved.

### 2.3 Detection of antibodies against LPAI viruses

The samples tested were serum samples, eggs samples or both. A commercial test kit, IDEXX FLockCheck AI MultiS‐Screen, for the detection of antibodies against avian influenza (all serotypes), was used for diagnosis. Test procedures for testing sera and prepared egg samples were those recommended by the manufacturer.

Egg samples were prepared for testing as follows: one ml of egg yolk was collected from each egg using a 1‐ml tip and pipette and loaded into a 5‐ml tube. An equal amount of 0.01 M PBS was added (dilution = 1/2), vortexed and centrifuged at 1,500 *g* for 30 min. The supernatant was collected and used for testing. In the ELISA test, the supernatant was further diluted fivefold with the kit's sample diluent. Thus, the final egg‐yolk dilution used in the assay was 1/10 (the same working dilution as for serum samples). Following the manufacture's recommendations, samples with an ‘absorbance sample/absorbance negative controls’ (S/N) value lower than 0.5 were categorized as positive. A study validating the use of egg‐yolk samples was performed elsewhere. This study showed a high concordance between the ELISA tests using serum and egg samples, with sensitivities of 98% and 99% for serum and egg samples, respectively, and specificities of 99% for both types of samples (Gonzales et al., 2012).

A possible source of bias in this study was the effect of storing eggs for periods up to 6 months on persistence of antibodies against AI and hence the performance of the test. Therefore, an experiment was performed where this effect was assessed (see Supplementary information S1 for detailed methods and results from this experiment).

### 2.4 Statistical analysis

#### 2.4.1 Estimation of the time of introduction of a LPAI infection in a flock

**Figure 1:**
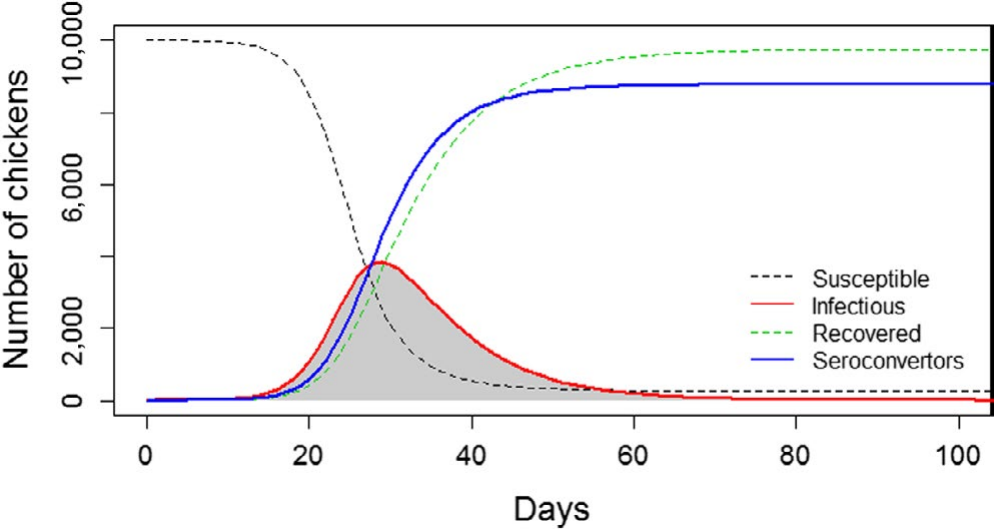
Low pathogenic avian influenza (LPAI) infection dynamics. A susceptible–infectious–recovered (SIR) model was used for this simulation. A transmission rate *β* = 0.49/day, recovery rate *α* = 1/7 days, seroconversion rate = 1/4 days and a probability of seroconversion = 0.9 were used for this simulation. Parameters' values were obtained from Gonzales et al. (2014)

In Figure 1, the infection dynamics of an outbreak of LPAI in chickens is shown. These dynamics were modelled using a deterministic susceptible–infectious–recovered (SIR) model (see Keeling and Rohani (2008) for a description of the differential equations). Based on this SIR model, the prevalence of seroconverting animals in time was also obtained as described elsewhere (Gonzales, Boender, Elbers, Stegeman, & de Koeijer, 2014). It can be seen (Figure 1) that the number of seroconverting chickens *C*_t_ in time *t* follows a sigmoidal behaviour. Therefore, this behaviour was modelled using a logistic growth curve where (Equation 1):

(1) $$C_{t}=\frac{F}{1+\left(\frac{F}{C_{0}}-1\right)\times e^{-rt}}.$$

In this model, *F* is the final size of the epidemic (total number of cases at the end of the epidemic),*C*_0_ is the number of seroconverting chickens at the start of the exponential growth and *r* is the growth rate which is equal to the transmission rate *β* minus the recovery rate $\alpha \left(r=\beta -\alpha \right).$

This model (Equation 1) was first validated by fitting it to simulated data (sampling 2 to sampling 4 prevalence estimates in time from the simulated outbreaks) with different transmission dynamics (Figure 1). Data were simulated using SIR models as described above. The model closely recreated the seroconversion curves of the simulated data (data not shown) and deviation from the real introduction time ranged from 1 to 10 days, with the largest deviation observed when few data values (only two) were available (data not shown). We considered this deviation as acceptable. Following validation, the model was fitted to seroprevalence data obtained from each infected farm, by testing either eggs or sera or both, during the study period. Model fits allowed the quantification of *r* and *C*_0_. These values were then used to estimate the time of introduction by solving for *t* when *C*_t_ = 1. This value either negative or positive represented the number of days before or after the last date the farm was negative when infection was expected to have entered the flock.

**Figure 2:**
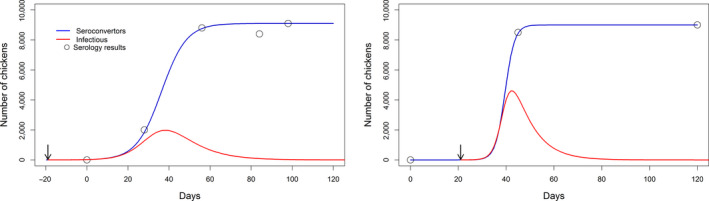
Reconstruction of low pathogenic avian influenza (LPAI) introduction time and outbreak dynamics within a flock using seroprevalence data. The blue line shows the result of the model fit (Equation 1) to the prevalence data (open circles), representing the prevalence of seroconverting chickens in time. The red line is the predicted dynamics (using an SIR model) of infectious animals based on the model estimates (Equation 1) and the arrow marks the estimated time of introduction. (a) This is a farm detected during serological surveillance (day 98) as positive for LPAI H6N2. Following detection, the prevalence in time was estimated retrospectively by testing stored egg samples. Time of introduction was around 47 days before the first positive tests (day = 28). (b) This farm was detected by serological surveillance as positive for LPAI H9N2. This farm was not part of the cohort study; hence, no egg samples were stored. Estimated introduction time for this outbreak was around 24 days before the first positive test

The following assumptions/steps were made in order to fit the model:

- The proportion of positives observed at each sampling point (total samples were mostly 30 eggs or sera samples) were considered to be a close approximation of the ‘real’ prevalence.
- For simplicity and due to data limitations, we did not consider the time from infection to seroconversion in serum or egg samples (Gonzales et al., 2012). It was assumed that seroconversion took place at the time of recovery (on average 7 days postinfection) for both serum and eggs.
- In case of farms (study period 2015–2016) where egg samples were tested longitudinally or serological history was well known, there were three to four seroprevalence observations. For these farms, both parameters *r* and *C*_0_ were quantified (Figure 2a).
- For farms where only one (first time positive) or two seroprevalence observations were available (study period 2013–2014), the value of *r* was sampled from an assumed range of values and only *C*_0_ was quantified by fitting the model to the data. The selected *C*_0_ value was that from the model with the lowest Akaike information criterion (AIC) (Figure 2b). Assumed values for *r* were based on i) the LPAI serotype of the detected introduction and ii) reported *β* and *α* values, which were reviewed for different LPAI serotypes (Central Veterinary Institute et al., 2017). For these farms, there was an interval between the first time positive and last time negative of 3 months and the model could not converge when using this long interval; therefore, we took half of these periods as the time at risk of the probable introduction and used therefore 1.5 months as the last time the prevalence was likely to be zero.
- An example code of the model and fitting process is provided as supplementary information (Supplementary Information S2). Model fitting was done using the nls function of the software package R (R Core Team, 2017).

#### 2.4.2 Assessing the temporal risk of LPAI introduction

Once the expected times of introduction were estimated, the results were grouped per calendar month together with the total number of farms sampled and tested each month. This information was then used to assess the seasonal risk for introduction of LPAI by fitting a harmonic regression model with a Poisson error distribution. This model explicitly includes time as a numeric covariate and characterizes seasonal patterns in terms of amplitude (ratio of the peak prevalence to the trough (minimum) prevalence) and phase shift (Stolwijk, Straatman, & Zielhuis, 1999). We evaluated whether seasonal patterns had a yearly cycle (one peak and trough per year [12‐month period]), semi‐yearly cycles (6‐month period) or a combination of both. The final model explained a yearly cycle (Equation 2)

(2) $$\log\mu =\alpha +\beta_{1}t+\beta_{2}t^{2}+\beta_{3}\sin\left(\frac{2\pi t}{12}\right)+\beta_{4}\cos\left(\frac{2\pi t}{12}\right)+\log\left(\mathrm{farm}\right).$$

where µ is the number of introductions, *α* is the model intercept, *t* is the month number within the study period (months 1–48) and this variable is used to assess the temporal trend in the number of introductions, and *β*_1_ and *β*_2_ are the parameters describing this trend. Finally, *β*_3_ and *β*_4_ are the parameters describing the seasonal yearly cycle (characterized by the pair of sine and cosine functions). These latter parameters were used to identify the periods of peak and minimum prevalence as well as the amplitude following formulas described elsewhere (Stolwijk et al., 1999). Finally, *farm* is the total number of farms sampled monthly and it was included as an offset. This analysis was done using the software package R (R Core Team, 2017).

Additionally, we assessed whether there were differences in the frequency of introductions among calendar months. To this end, we fitted a generalized mixed model (GLMM) where year was used as a random effect and month as a categorical variable, with ‘June’ used as the reference month for comparison. Because multiple pair (between months) comparison was made (*n* = 11), a Bonferroni correction was used to determine significant differences (*p* < .05/11) and reduce the risk of false‐positive errors (observing significant results when they are actually not significant).

#### 2.4.3 Time of introduction of a HPAI infection in a flock

HPAI H5N8 group A and group B viruses were introduced in the Netherlands in the winter of 2014–2015 and 2016–2017. In 2017–2018, the HPAI H5N6 group B virus was introduced. The HPAI viruses were introduced in different poultry species (chickens, ducks) and holding facilities (indoor and outdoor laying hens, breeding farms). Tracheal and cloacal swabs from clinically affected poultry were tested in a matrix‐gene real‐time PCR and subtyped using H5‐specific real‐time PCR as previously described (Bouwstra et al., 2015). The sequence of the HA cleavage site and the N‐subtype was determined by the Sanger sequencing. We previously analysed the complete genome sequences of the viruses detected at the farms, and performed genetic analysis to identify the source of infection (Beerens et al., 2019; Bergervoet et al., 2019; Bouwstra et al., 2015). Included in this study are farms infected by primary virus introductions from wild birds, whereas farms likely infected by secondary farm‐to‐farm transmission of the virus were excluded. We have previously shown that HPAI introductions are likely detected within 7–12 days following introductions (Bos et al., 2007; Gonzales & Elbers, 2018).

## 3 RESULTS

### 3.1 Detection of antibodies against LPAI viruses

Blood samples or stored eggs collected at free‐range chicken farms were analysed for the presence of antibodies against LPAI viruses. An experiment was performed to assess antibody persistence in stored eggs, which showed no significant changes in antibody persistence and diagnostic outcomes for the first 3 months of storage (Supplementary information S1). Therefore, no influence in the study was expected given that farms were serologically sampled every 3 months and egg samples tested from surveillance‐detected seropositive farms were not stored longer than this period.

Data on total number of free‐range layer farms sampled as part of the monitoring programme and number of LPAI introductions are presented in Table 1. During the prospective study, a total of 20 seropositive farms were detected, 12 of these farms participated in the cohort study and stored eggs from these farms were tested. For all these farms, information on the last time the farm was negative, and the first time positive and changes in seroprevalence (% positives) in time (when egg or sera samples were available) were recorded (see example provided in Supplementary information S2). The remaining eight introductions in the period 2015–2016 and those from the retrospective study (*n* = 57) were detected during routine surveillance.

**Table 1:** Number of tests (one test = one farm) made and total number of low pathogenic avian influenza (LPAI) introductions analysed for each year of the study period

Year	Farms	Introductions
2013	1,813	29
2014	1,912	28
2015	1,931	14
2016	1,961	6
Total	7,617	79

### 3.2 Estimating the time of LPAI introduction

Time of introduction was estimated by fitting a mathematical model to seroprevalence data collected longitudinally from infected farms. Figure 2 shows examples of the model fitted to prevalence estimates for a farm using egg and serum samples (Figure 2a) or a farm with information from serum samples only (Figure 2b). In Figure 2a, the outbreak dynamics for a flock affected with a H6N2 LPAI virus are recreated. Available seroprevalence data allowed estimation of both the growth rate *r* parameter and *C*_0_. The estimated time of introduction was approximately 47 days before the eggs tested positive. In Figure 2b is shown the dynamics for a flock affected with a H9N2 LPAI virus. Data were limited, and hence, only one parameter, *C*_0_, could be estimated and *r* had to be assumed. We assumed that the H9N2 virus spreads rapidly within a flock based on previous results (Central Veterinary Institute et al., 2017). The estimated time of introduction of this virus was around 24 days before the first positive result. Full results and analysis code for these figures are given as Supplementary Information S2.

### 3.3 Temporal (monthly) risk of LPAI introduction

**Figure 3:**
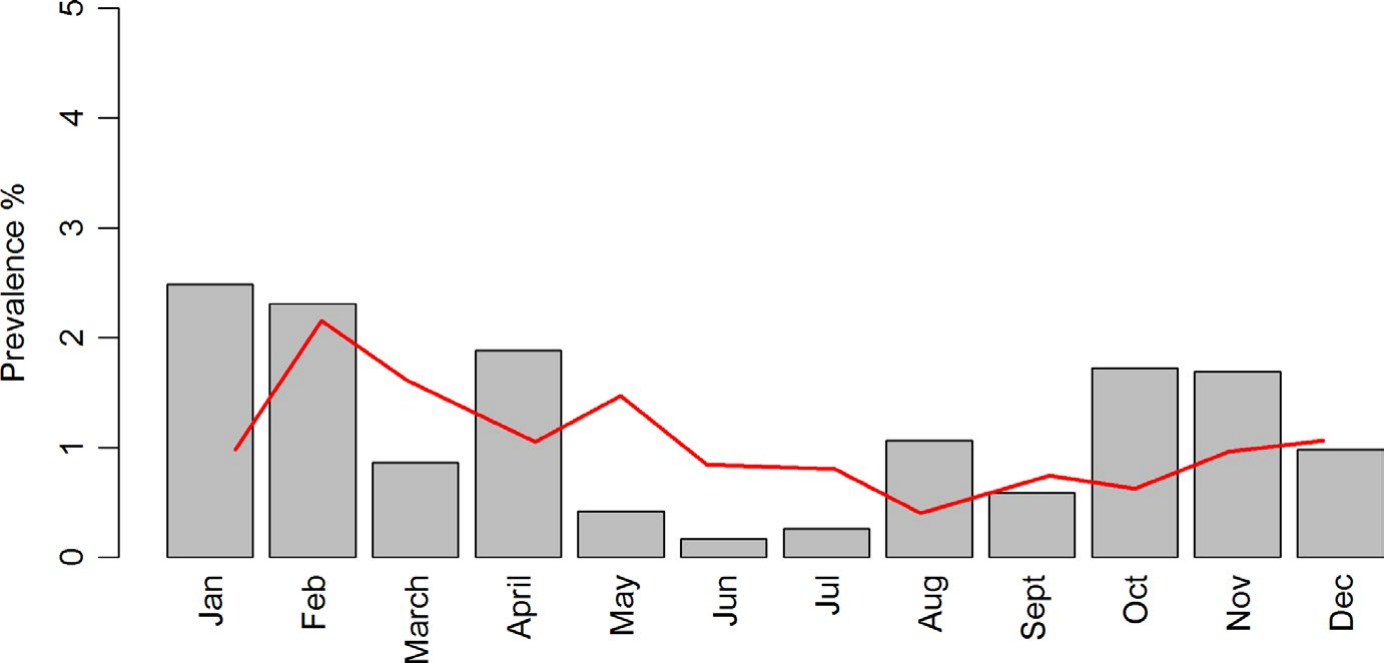
Monthly prevalence (in %) of low pathogenic avian influenza (LPAI) introductions (bars) as estimated using the model described in Equation (1) or serological positive detections (based on the date of detection within the surveillance system). Farm population is limited to free‐range farms and the study period 2013–2016

Following the estimation of introduction times for each of the seropositive flocks included in this study, we aggregated the data at monthly level. Figure 3 shows the average monthly prevalence (%) of introductions and serological detections for the study period (2013–2016). A GLMM was used to make an overall comparison of the frequency of introductions per month. June was used as reference, and the months identified as significantly different (*p* < .0045) from June were November, January and February.

**Figure 4:**
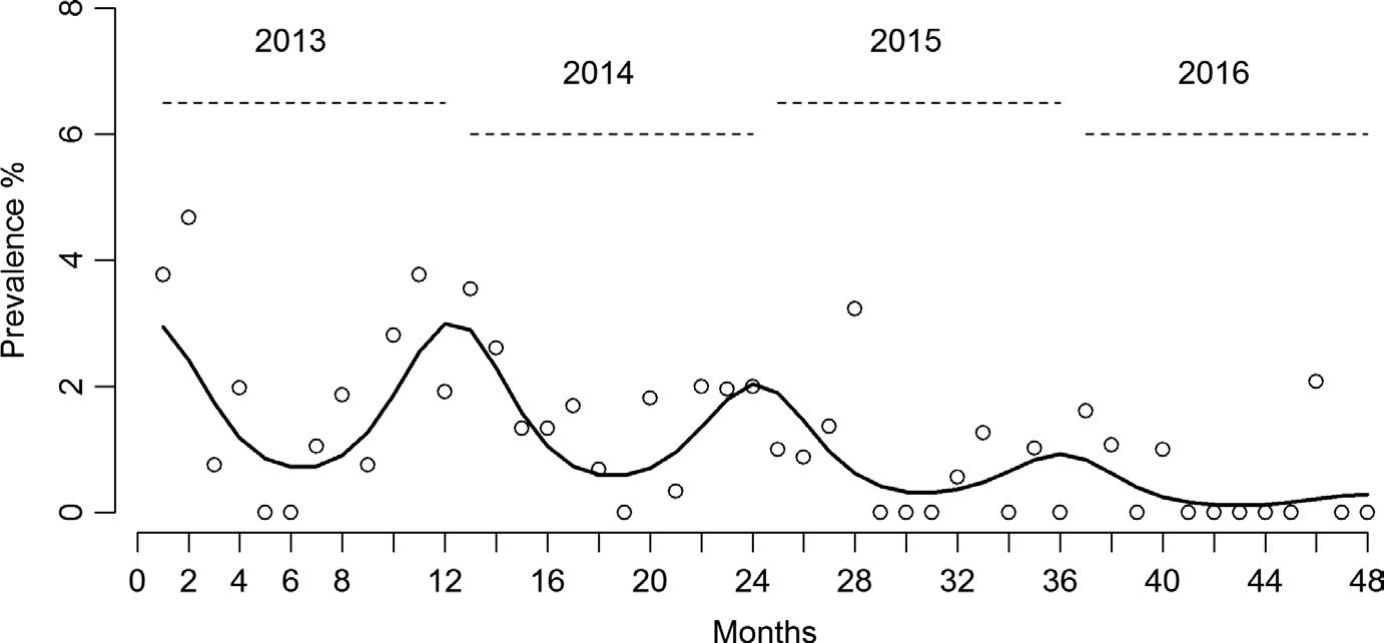
Seasonal variation in the risk for introduction of low pathogenic avian influenza (LPAI) in outdoor layer farms

The results of the harmonic regression model showed a consistent drop in the prevalence of introductions along the years and presence of a significant yearly cycle (seasonality) with a peak prevalence of introductions in January and the lowest prevalence of introductions in June (Figure 4). The average relative risk (RR) between the peak period relative to the lowest period was 4.5 (95% confidence limits [CL)]: 2.2–9.2) and the duration of the peak cycle (number of months at highest risk) was on average 4.2 (95% CL: 3.7–6.0) months, between November and February.

### 3.4 Temporal (monthly) risk of HPAI introduction

**Figure 5:**
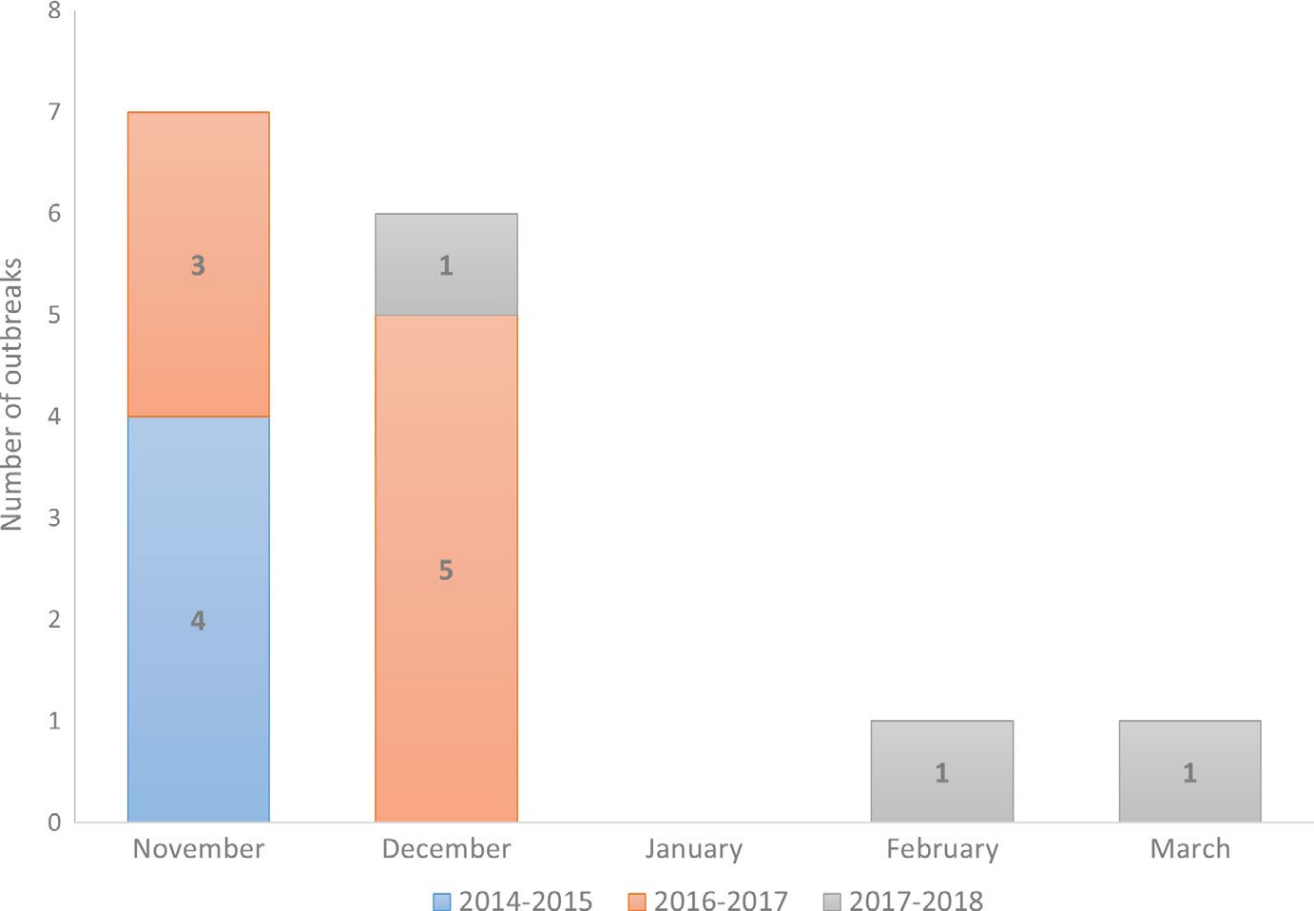
Number of HPAI introductions in poultry (commercial flocks) aggregated by month of introduction during the period from 2014 to 2018

We qualitatively assessed the temporal risk of HPAI introductions in commercial poultry flocks by monthly aggregating the detections of HPAI H5 viruses between 2014 and 2018. HPAI introductions are likely detected within 7–12 days following introduction of the virus. The observed period of high risk for introduction of LPAI appears to correlate with the period when most HPAI introductions in poultry were observed in the Netherlands. Most HPAI introductions were observed in the months of November and December (Figure 5).

## 4 DISCUSSION

The main objective of this study was to assess whether there is a temporal risk for introduction of LPAI infections in free‐range layer farms. This temporal risk was confirmed: a significant yearly cycle was observed with the highest risk expected around January and the lowest risk around June (Figures 3 and 4). The high‐risk period for introductions has an average duration of 4 months, between November and February (Figure 4). Additionally, we qualitatively identified a high‐risk period for introduction of HPAI infections in poultry (Figure 5). This period appears to be similar to the risk period for LPAI virus introductions identified for free‐range layers and together might indicate that this risk period may apply to most poultry production types in the Netherlands.

The apparent similarity in the period of highest risk for introductions of LPAI and HPAI virus in poultry (particularly free‐rage layers) provides further evidence that the main source of introductions for LPAI could also be migratory waterfowl, particularly species of the order Anseriformes (ducks, geese and swans) (European Food Safety Authority et al., 2017). In fact, the identified high‐risk period for introduction of LPAI in free‐range layers appears to correlate with the period (2015–2016) of higher abundance of migratory birds such as, for example, Eurasian wigeons (*Mareca penelope*), tufted ducks (*Aythya fuligula*) and geese and swan species in general (Hornman et al., 2018). Additionally, a study in the Netherlands that video‐monitored wildlife visits to a free‐range farm—which is located in a region of high wild bird abundance and suffered several introductions of LPAI viruses over different years—observed a relative temporal increase in the number of visits (birds) by dabbling ducks in the period between December and February, whilst the periods of highest visits of members of the order Charadriiformes and Passeriformes were April–July and June–September, respectively (Elbers & Gonzales, 2019). Despite this overlap in the temporal risk of introduction and Anseriformes abundance, the question remains whether the same or different wintering (migratory) species arriving to the Netherlands are implicated in the introduction of both LPAI and HPAI (Hornman et al., 2018; Kleyheeg et al., 2017; Verhagen et al., 2017).

Despite the intensive surveillance programme performed in the Netherlands and the large amount of farms investigated thereby (Bouwstra et al., 2017; Gonzales et al., 2010), quantitative characterization of the temporal risk for introduction of LPAI viruses has been challenging (Verhagen et al., 2017). This difficulty arises mainly because the prevalence of introductions of LPAI virus in poultry is low and variable from year to year (Bouwstra et al., 2017; Gonzales et al., 2013); hence, high sample sizes and sampling frequency are required to confidently assess seasonality. This low prevalence and yearly variability influenced our study. The number of observed introductions during the prospective study (despite intensive sampling and large sample size) was low (*n* = 20) and was not enough to assess seasonality as originally intended; hence, we had to include serological surveillance data from previous years, which was not as detailed (temporal information on prevalence) as the data generated from the prospective study. Therefore, in order to use these data, a number of assumptions had to be made (see Section 2.4, 3.1). An influential assumption that is worth noting is that of the assumed transmission dynamics of LPAI viruses. This assumption has a direct influence on the estimated introduction time. We based our assumption on the LPAI virus HA subtype and the prevalence at the assumed end of the epidemic (final size). For example, we assumed that all H9 viruses found in our study had similar transmission characteristics to those reported for a LPAI H9N2 virus isolate (Central Veterinary Institute et al., 2017). However, the transmission characteristics of a virus are not determined by the HA only, and other viral genes and specific genome characteristics may play a role. For instance, it has been shown that different H7 serotypes can vary considerably in transmissibility (Central Veterinary Institute et al., 2017; Gonzales et al., 2014). The mechanisms (e.g. genetic traits) influencing the transmissibility of LPAI virus are not yet understood and understanding them as well as identifying some genetic markers for virulence and/or transmissibility (Baron et al., 2013; Shaib et al., 2011) will lead to the swift assessment of the transmission risk of detected LPAI viruses introductions in poultry and the implementation of suitable control measures. In summary, the limited data availability and the assumptions made for the estimation of the time of introduction are important limitations of this study.

The quantified duration and relative risk period can be used to optimize surveillance for LPAI by targeting serological sampling of free‐range layers during the identified risk period. Currently, all free‐range layer farms are sampled every 3 months and sampling is organized in a way that a similar number of samples are taken monthly along the year. Based on the results of this study, sampling can be re‐organized so that frequency of sampling could be increased during the high‐risk period (e.g. every 2 months (Gonzales et al., 2014)) and reduced during the low‐risk period. Additionally, sampling of other high‐risk poultry species (domesticated ducks and turkeys) can be targeted around this period too. These changes in sampling strategy will improve timelines of detection and probably improve surveillance sensitivity whilst maintaining the current sampling costs. Knowledge of the seasonal characteristics can also guide the implementation or enhancement of preventive measures during the risk period. Measures targeted to reduce or prevent direct or indirect contact between chickens and wild birds could be enhanced in this period. Some of these measures could be the prevention of water pool forming in the free‐range area (improved drainage or equalizing the soil), increased frequency of collection of eggs and carcases from the free‐range area (Elbers & Gonzales, 2019) and use of for example trained dogs (Castelli & Sleggs, 2000) or laser technology to prevent wild birds visiting the free‐range area of a poultry farm (Scott & Clark, 2006). Finally, the identified risk period could also guide the implementation of indoor confinement of free‐range layers.

Despite the increase in the number of free‐range layer farms in the Netherlands, which increases the opportunities for contact between poultry and wild birds, thereby increasing the risks for AI introductions (Bouwstra et al., 2017), a decreasing trend in the prevalence of introductions of LPAI was observed (Figure 4). We cannot explain the reasons of these decreasing trends. We explored whether changes in weather such as temperature and rainfall could be associated (indirectly) with this trend, but we did not find any significant differences in these weather variables (data not shown) during the study period (years 2013 to 2016). We hypothesize that the observed trend and seasonality of introductions could be associated with changes in migratory patterns of some wild bird species likely to play a significant role in the introduction of AI in poultry (still unknown). An approach to answer this question could be a spatiotemporal analysis where the time and space relationship between the distribution of AI introductions in poultry and abundance of different wild bird species is assessed. We are, in collaboration with ornithologist, currently performing such studies.

The results of this study are mainly applicable to the Dutch situation and to free‐range layers in particular. We do not know whether the identified risk period, or presence of a distinct seasonal risk, would be similar in other European countries. The correlation observed between the risk period of introductions in poultry and the period of higher abundance of migratory waterfowl in the Netherlands might be used as correlate (abundance of migratory waterfowl) of seasonal risk that could be used to identify potential risk periods in neighbouring countries.

To summarize, the results of this study can be used to optimize risk‐based surveillance, take preventive measures in high‐risk months and inform decisions on timing and duration of indoor confinement during AI outbreaks in the Netherlands. Reduction in AI introductions into commercial poultry will benefit animal welfare, economy and public health.

## CONFLICT OF INTEREST

The authors declare no conflict of interest.

## ETHICAL APPROVAL

The authors confirm that the ethical policies of the journal, as noted on the journal's author guidelines page, have been adhered to. No ethical approval was required as this study used data that have been collected under the compulsory AI surveillance programme in the Netherlands (Bouwstra et al., 2017) or have been (HPAI data) previously reported (Beerens et al., 2019; Bergervoet et al., 2019; Bouwstra et al., 2015). New generated data come from the egg samples (non‐invasive sampling), which were obtained following consent of the farmers who joined the prospective study.

## Supporting information

Persistence of antibodies against avian influenza in eggs

Example code and data for estimating the time of introduction using seroprevalence data

## Data Availability

The data that support the findings of this study are available from the corresponding author upon reasonable request.
