# Supplementary material for: Seasonal risk of low pathogenic avian influenza virus introductions into free‐range layer farms in the Netherlands
Source: Transbound Emerg Dis. 2020 Jun 7;68(1):127–36. doi: 10.1111/tbed.13649 (PMC8048991; doi:10.1111/tbed.13649)
Supplement: Supplementary file 1 — Persistence of antibodies against avian influenza in eggs [file TBED-68-127-s001.docx]

**Persistence of antibodies against avian influenza in eggs**

1. **Egg samples and laboratory methods**

A total of 420 egg samples were randomly taken from a LPAI seropositive flock at the beginning of the study. From these egg samples, 60 eggs were selected at random every testing month for a period of six months (7 tests). Egg yolk samples were prepared for testing as described in the main manuscript and by (Gonzales 2012) and tested using the haemaglutination inhibition (HI) test following procedures described in the OIE manual, using eight haemagglutination units (HAU) of antigen H8N4 A/Ty/Ontario/6118/67 (OIE, 2017). Test conditions were standard and were carried out at the same lab and by the same scientist.

Low antibody titres were expressed as < 3 (log_2_ transformed). For these specific set of observations, assumptions have to be made about its possible values and their inclusion in the analysis. Two assumptions were made: 1) these observations were given a fixed value equal to 2.5, 2) This observations were considered ‘negative’ and excluded from the analysis.

1. **Data analysis**

Data for each testing month were first assessed for normality using the Shapiro-Wilk test. Data were not normally distributed. However, given that the sample size each month is large (n = 60) the assumption of normality could be relaxed. Therefore, analysis were done using parametric methods as screening test and later confirmed by non-parametric methods as follow:

1. Graphical inspections were done to summarise the data, evaluate assumptions and identify potential differences in titres between different testing months
2. As a fast screening: quantified antibody titres at each month of testing following egg storage were compared by analysis of variance and multiple comparisons between months were made using Tukey’s test.
3. To confirm identified significant differences, the Kruskal-Wallis test was carried out with all groups included. Then between group comparisons (two-group) of medians were done with the Wilcoxon test.
4. The threshold of significance was adjusted using Bonferroni’s correction: 0.05/21 = 0.0023. Where 21 is the number of multi-group comparisons
5. **Results**

Summary mean titres for assumptions 1 and 2 are shown in table 1 and Figure 1. Additionally the proportion of positives (titres > 3) each month are also presented. There are many observations with titres < 3 (classified as negative) during the first three months (Figure 1) and their inclusion (assigning values of 2.5) significantly reduces the mean titres these months compared to the last months.

**Table 1** Mean antibody titres against AI quantified in eggs stored from up to six months post sampling

|  | All data | | Excluding negatives (titre < 3) | |
| --- | --- | --- | --- | --- |
| Month | Mean | SD | Mean | SD |
| 1 | 3.48 | 1.41 | 5.36 | 0.97 |
| 2 | 3.81 | 1.55 | 6.32 | 0.77 |
| 3 | 3.46 | 1.24 | 6.82 | 1.42 |
| 4 | 4.32 | 1.25 | 6.2 | 0.92 |
| 5 | 4.43 | 1.09 | 5.86 | 0.89 |
| 6 | 4.87 | 0.83 | 6.17 | 0.92 |
| 7 | 4.98 | 1.33 | 6.8 | 0.86 |

Given the unknown condition of the eggs with respect to antibody presence and levels against AI, it was not possible to confidently identify whether the reason for the difference in proportion of samples with titres higher >3 (classified as positive) between the first three months and the last four (Figure 1) was due to sampling or effect of the long storage of eggs. Therefore comparison were done for both assumptions.


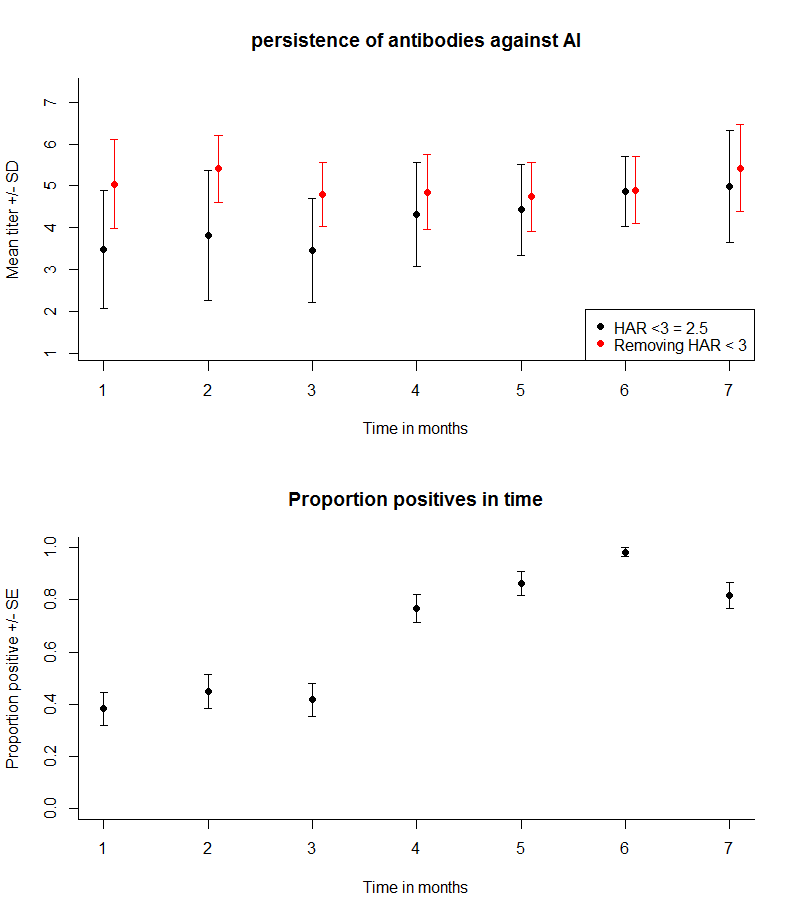


Figure 1. Mean antibody titres (upper figure) and proportion (bottom figure) of samples with HI (HAR) titres > 3. Month 1 is the starting date or time 0.

Table 2 shows the results of the comparison (ANOVA-Tukey tests) of antibody titres between each month of evaluation. Significant differences were confirmed only between months 5 and 7 (Wilcoxon test, p = 0.0005).

The results of comparisons made using all the data (including data with titres values < 3) are given in Table 3. Statistical differences (confirmed also with the Wilcoxon test) were observed between the first three months and the last three. No difference between months 1 to 4 were observed.

**Table 2** Comparison of antibody titres between each month excluding negatives egg samples (titre < 3). In bold months that differ significantly, which were confirmed by the Wilcoxon Test (p < 0.0023)

| **Months** | **Diff*** | **LWR** | **UPR** | **P** |
| --- | --- | --- | --- | --- |
| 2-1 | 0.36393 | -0.38445 | 1.11231 | 0.77700 |
| 3-1 | -0.24348 | -1.00551 | 0.51855 | 0.96403 |
| 4-1 | -0.19565 | -0.86920 | 0.47789 | 0.97763 |
| 5-1 | -0.30348 | -0.96798 | 0.36103 | 0.82414 |
| 6-1 | -0.14517 | -0.79351 | 0.50317 | 0.99433 |
| 7-1 | 0.38509 | -0.28154 | 1.05173 | 0.60593 |
| 3-2 | -0.60741 | -1.33945 | 0.12463 | 0.17647 |
| 4-2 | -0.55958 | -1.19900 | 0.07984 | 0.13013 |
| 5-2 | -0.66741 | -1.29730 | -0.03752 | 0.02990 |
| 6-2 | -0.50910 | -1.12191 | 0.10371 | 0.17531 |
| 7-2 | 0.02116 | -0.61097 | 0.65330 | 1.00000 |
| 4-3 | 0.04783 | -0.60751 | 0.70316 | 0.99999 |
| 5-3 | -0.06000 | -0.70604 | 0.58604 | 0.99996 |
| 6-3 | 0.09831 | -0.53110 | 0.72771 | 0.99924 |
| 7-3 | 0.62857 | -0.01966 | 1.27681 | 0.06406 |
| 5-4 | -0.10783 | -0.64666 | 0.43101 | 0.99694 |
| 6-4 | 0.05048 | -0.46829 | 0.56925 | 0.99995 |
| 7-4 | 0.58075 | 0.03928 | 1.12221 | 0.02660 |
| 6-5 | 0.15831 | -0.34867 | 0.66528 | 0.96787 |
| **7-5** | **0.68857** | **0.15840** | **1.21875** | **0.00270** |
| 7-6 | 0.53027 | 0.02050 | 1.04003 | 0.03547 |

* Diff = difference between means. LWR, UPR: lower and upper limits of the difference

**Table 3** Results of the analysis of variance using all observations. In bold months that differ significantly, which were confirmed by the Wilcoxon Test (p < 0.0023).

| **Month** | **diff*** | **LWR** | **UPR** | **P** |
| --- | --- | --- | --- | --- |
| 2-1 | 0.3333 | -0.3501 | 1.0168 | 0.7768 |
| 3-1 | -0.0167 | -0.7001 | 0.6668 | 1.0000 |
| 4-1 | 0.8417 | 0.1582 | 1.5251 | 0.0055 |
| **5-1** | **0.9560** | **0.2667** | **1.6454** | **0.0009** |
| **6-1** | **1.3917** | **0.7082** | **2.0751** | **0.0000** |
| **7-1** | **1.5083** | **0.8249** | **2.1918** | **0.0000** |
| 3-2 | -0.3500 | -1.0335 | 0.3335 | 0.7343 |
| 4-2 | 0.5083 | -0.1751 | 1.1918 | 0.2957 |
| 5-2 | 0.6227 | -0.0666 | 1.3120 | 0.1068 |
| **6-2** | **1.0583** | **0.3749** | **1.7418** | **0.0001** |
| **7-2** | **1.1750** | **0.4915** | **1.8585** | **0.0000** |
| 4-3 | 0.8583 | 0.1749 | 1.5418 | 0.0042 |
| **5-3** | **0.9727** | **0.2834** | **1.6620** | **0.0007** |
| **6-3** | **1.4083** | **0.7249** | **2.0918** | **0.0000** |
| **7-3** | **1.5250** | **0.8415** | **2.2085** | **0.0000** |
| 5-4 | 0.1144 | -0.5750 | 0.8037 | 0.9990 |
| 6-4 | 0.5500 | -0.1335 | 1.2335 | 0.2078 |
| 7-4 | 0.6667 | -0.0168 | 1.3501 | 0.0612 |
| 6-5 | 0.4356 | -0.2537 | 1.1250 | 0.4998 |
| **7-5** | 0.5523 | -0.1370 | 1.2416 | 0.2123 |
| 7-6 | 0.1167 | -0.5668 | 0.8001 | 0.9988 |

* Diff = difference between means. LWR, UPR: lower and upper limits of the difference

**4. Conclusion**

The results of this assessment show that antibodies persist in egg samples for long periods of time and no significant changes in titres were observed during the first three months of storage.

**References**

Gonzales, J. L., A. R. W. Elbers, J. A. Stegeman, W. G. Buist, G. Koch, S. de Wit, A. Bouma and B. Engel, 2012: Probability of serconversion against low pathogenic avian influenza virus infections in chickens and time to antibody detection in sera and egg samples. Surveillance of low pathogenic avian influenza in layer chickens: risk factors, transmission and early detection (PhD thesis), p. 167. Utrecht University, Utrecht.

OIE (2017), Chapter 2.3.4. Avian Influenza (infection with avian influenza viruses), Manual of Diagnostic Tests and Vaccines for Terrestrial Animals, France, 2017.
