# Supplementary material for: Seasonal risk of low pathogenic avian influenza virus introductions into free‐range layer farms in the Netherlands
Source: Transbound Emerg Dis. 2020 Jun 7;68(1):127–36. doi: 10.1111/tbed.13649 (PMC8048991; doi:10.1111/tbed.13649)
Supplement: Supplementary file 2 — Example code and data for estimating the time of introduction using seroprevalence data [file TBED-68-127-s002.docx]

Supplementary information 2.

**Example code and data for estimating the time of introduction using seroprevalence data**

## Function to plot logistic growth curve --------------

plotlog <- function(f,c0,r,k,mint,maxt){

# f = final size or prevalence at the end of the epidemic

# c0 = parameter for the initial exponential growth

# r = grow rate parameter

# k = total number of chickens in the flock

# mint, maxt = minimum and maximum time in days. Limits of the x axis

curve(

f*(k/(1+((k/c0)-1)*exp(-r*x))),

from=mint,

to=maxt,

xlab='Days',

ylab='Number of chickens',

col='mediumblue',

ylim = c(0,k),

lwd = 2,

cex.lab = 1.3

)

}

### estimate introduction date using serology data ---------

## Figure 2a --------

# Data --------

# virus serotype H6N2

time <- c(0,28,56,84,98) # time in days (intervals between serological tests)

# dates serological test: 15-12-2015 (egg), 12-1-2016(egg), 9-2-2016 (egg), 8-3-2016 (egg),

# 22-3-2016 (sera)

d_neg <- as.Date("15/12/2015", "%d/%m/%Y") # date last negative

prev <- c(0,0.2,0.88,0.84,0.91) # estimated prevalence at each testing date. Last value is the final size

sero <- 10000 * prev # Number of infected chickens for a flock of 10000 birds

lp2 <- data.frame(time,sero) # join time and sero in a dataframe

# Fit model to estimate parameters c0 and r

fit2 <- nls(

sero~max(sero)/(1+((max(sero)/c0)-1)*exp(-r*time)),

lp2,

start=c(c0 = 1, r = 0.2),

trace=F

)

summary(fit2)

# day (date) of introduction

d_intro <- (log(1)-log(coef(fit2)[1]))/coef(fit2)[2]

Date_neg <- d_neg # date last negative

Date_intro <- d_neg + d_intro # date of introduction

# plot results

plotlog(max(prev),coef(fit2)[1], coef(fit2)[2],10000,d_intro,120)

points(lp2$time,lp2$sero, cex = 2)

## Figure 2b -------------------------------------

# H9N2 virus

## Assumptions

# high betas: 0.4 – 0.7

# recovery rate = 1/7.5

#Data

d_neg <- as.Date("20/01/2013", "%d/%m/%Y") # assumed intro within 45 days before first positive

time <- c(0,45,120)

prev <- c(0,0.85,0.9)

sero <- 10000 * prev

lp2 <- data.frame(time,sero)

r <- seq(0.4,0.5,0.01) # assumed values for r

C <- NULL

b <- NULL

# Fit model to quantify parameter c0

for(i in 1:length(r)){

fit2 <- nls(

sero~max(sero)/(1+((max(sero)/c0)-1)*exp(-r[i]*time)),

lp2,

start=c(c0 = 0.011),

trace=F

)

C[i] <- coef(fit2)[1] # c0 fitted values

b[i] <- AIC(fit2)

}

result <- as.data.frame(cbind(r,C,b))

# select model with lowest AIC

m_low <- which.min(result$b)

result[m_low,]

# day (date) of introduction

d_intro <- (log(1)-log(result$C[m_low]))/result$r[m_low]

D_0 <- d_intro

Date_neg <- d_neg # date last negative

Date_intro <- d_neg + d_intro

# plot results

plotlog(max(prev),result$C[m_low], result$r[m_low],10000,0,120)

points(lp2$time,lp2$sero, cex = 2)
